# BRAF V600E–Mutant Diffuse Pulmonary Langerhans Cell Histiocytosis Successfully Treated With Dabrafenib Plus Trametinib: A Case Report

**DOI:** 10.1002/rcr2.70339

**Published:** 2025-09-04

**Authors:** Akiko Sakurai, Tetsutaro Nagaoka, Chisato Tadokoro, Shunichi Kataoka, Yuriko Terayama, Yuichi Nagata, Yoshifumi Suzuki, Taichi Miyawaki, Motoyasu Kato, Takuo Hayashi, Kazuhisa Takahashi

**Affiliations:** ^1^ Department of Respiratory Medicine Juntendo University Graduate School of Medicine Tokyo Japan; ^2^ Department of Human Pathology Juntendo University Graduate School of Medicine Tokyo Japan

**Keywords:** BRAF V600E mutation, dabrafenib, pulmonary hypertension, pulmonary langerhans cell histiocytosis, trametinib

## Abstract

Pulmonary Langerhans cell histiocytosis (PLCH) is a rare diffuse lung disease that is strongly associated with cigarette smoking, with the BRAF V600E mutation identified in approximately half of all cases. In Japan, combination therapy with BRAF and MEK inhibitors has recently been approved as an alternative treatment option. We report the case of a 30‐year‐old woman diagnosed with BRAF V600E–mutant PLCH who was treated with dabrafenib in combination with trametinib. Chest computed tomography performed 3 months after the initiation of therapy demonstrated improvement in pericystic opacities, and this radiological response was sustained for over 1 year. Improvements in exercise tolerance, pulmonary function, and pulmonary haemodynamics have been observed clinically. These findings suggest that dabrafenib plus trametinib is a promising therapeutic option for patients with PLCH.

## Graphical abstract

We report the case of a 30‐year‐old woman diagnosed with BRAF V600E–mutant pulmonary Langerhans cell histiocytosis who was treated with dabrafenib in combination with trametinib. Chest computed tomography performed 3 months after the initiation of therapy demonstrated improvement in pericystic opacities. Both radiological and symptomatic recovery were sustained for over 12 months.

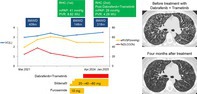

## 1 Introduction

Pulmonary langerhans cell histiocytosis (PLCH) is an “inflammatory myeloid neoplasm” characterised by clusters of atypical langerhans like cells that are positive for pathological markers CD1a, CD207 and S100 [1]. Almost all patients have a history of smoking [2]. PLCH most commonly occurs in patients aged 30–40 years, and its overall prevalence is low (0.27 per 100,000 in men and 0.07 per 100,000 in women). The prognosis is relatively favourable, with a 10‐year survival rate exceeding 90% [3, 4, 5]. Computed tomography (CT) reveals multiple cystic lesions and small nodules in the lung parenchyma. Smoking cessation is an important therapeutic strategy. Corticosteroids and/or immunosuppressants have been used to treat patients with disease progression even after quitting smoking; however, there is insufficient evidence for these treatments.

A recent report indicated that the BRAF V600E‐mutant has been detected in 57% of LCH cases, implicating it in disease pathogenesis [6]. Combination therapy with dabrafenib plus trametinib, a BRAF and MEK inhibitor respectively, has been approved in several countries. Here, we report a case of BRAF V600E‐positive PLCH treated with BRAF/MEK inhibitors, which improved the imaging findings and respiratory and circulatory functions.

## 2 Case Report

**FIGURE 1:**
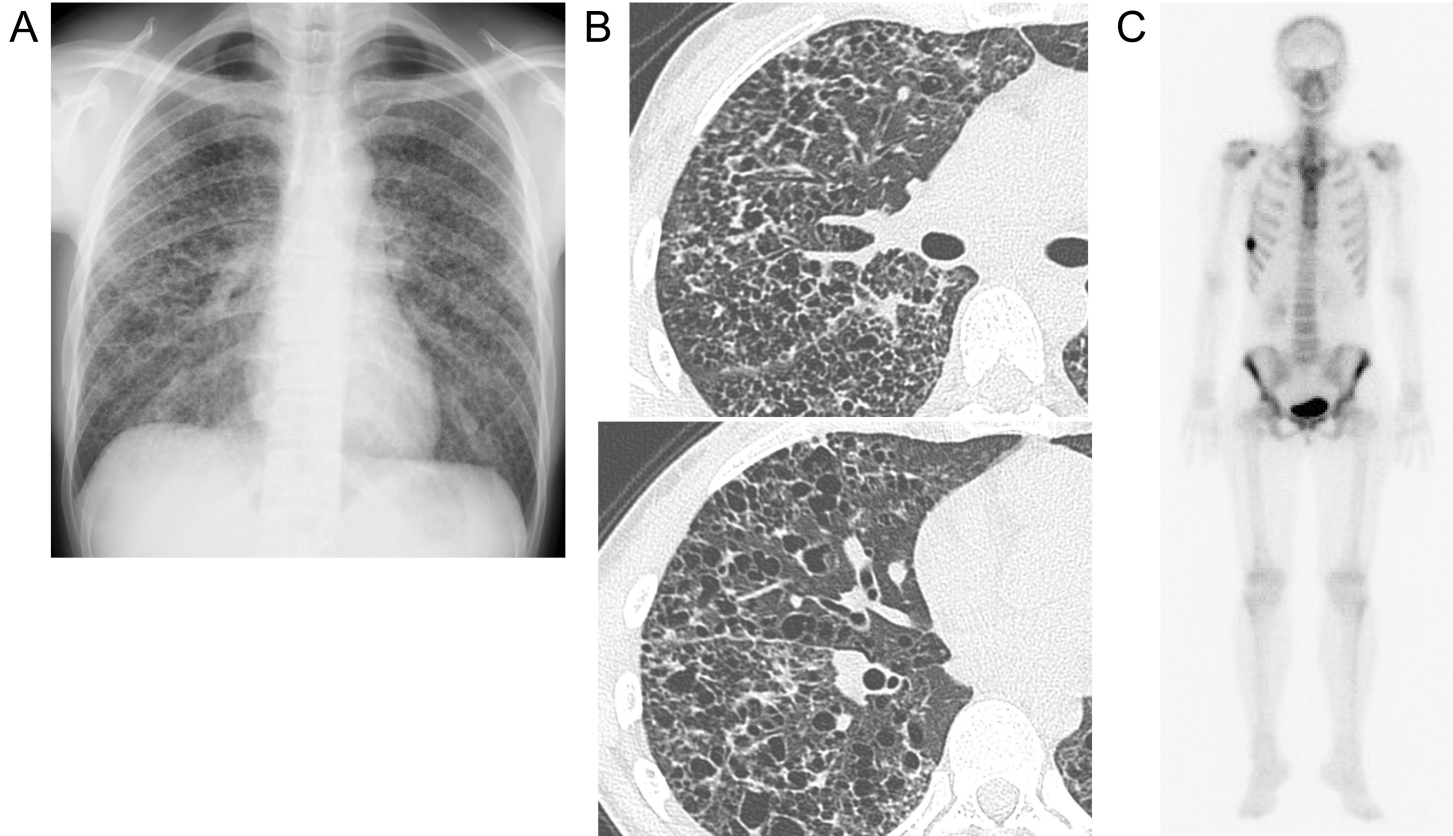
Chest radiograph showing bilateral diffuse ground‐glass opacities (A) Chest CT showing multiple bilateral cystic lesions with granular shadows (B) Brain MRI showed no intracranial lesions and mass‐like uptake in the right 10th rib on bone scintigraphy (C).

**FIGURE 2:**
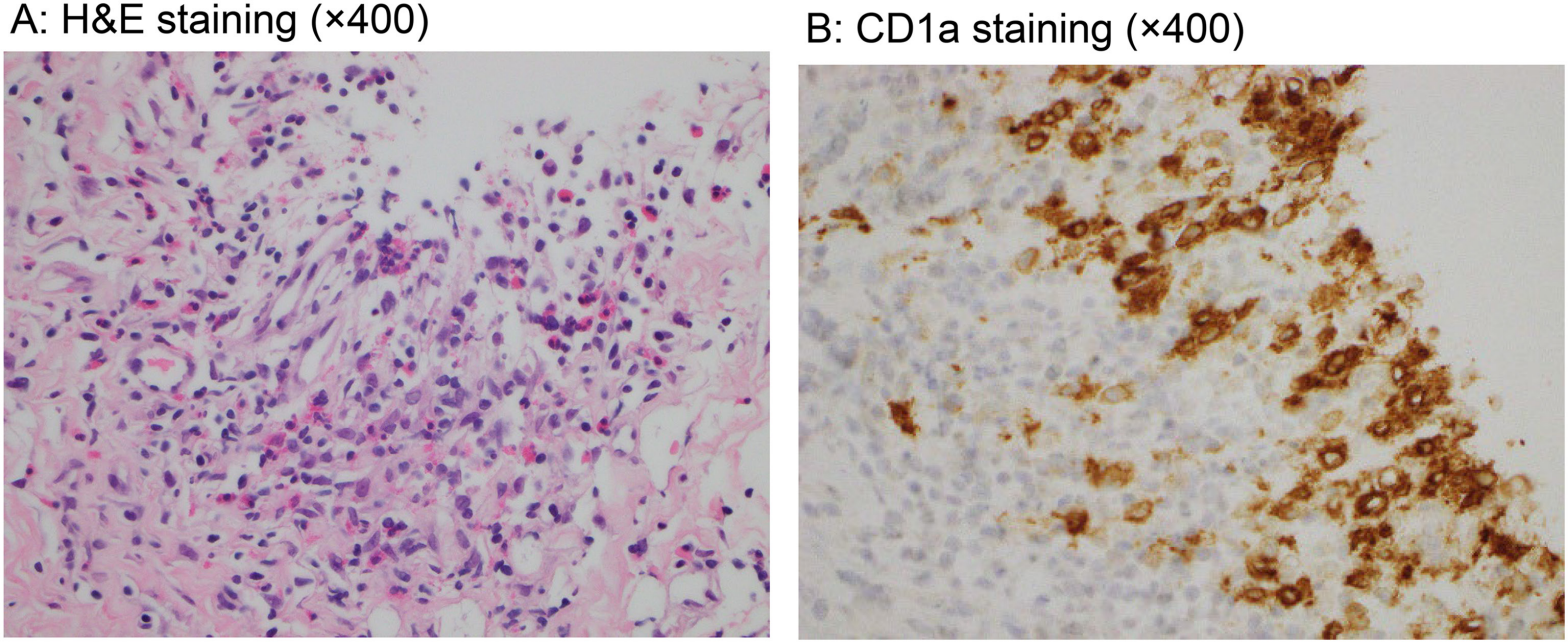
LCH cells and surrounding eosinophil infiltration (A) and those cells that were positive with CD1a (B) H&E, Haematoxylin and Eosin.

A 37‐year‐old woman with no medical history had dyspnea on effort for 4 years and visited the hospital in May 2021. She had a 10 pack‐year smoking history from the ages of 14 to 33 years. Chest radiography demonstrated bilateral diffuse ground‐glass opacity (Figure 1A), and chest CT revealed irregular, diffuse cystic lesions predominantly distributed in the upper lobes. The cyst walls appeared relatively thick and were accompanied by scattered granular opacities (Figure 1B). Based on these CT findings, lymphangioleiomyomatosis, sarcoidosis, and Birt‐Hogg‐Dubé syndrome were considered as differential diagnoses; however, PLCH was regarded as the most likely diagnosis according to the characteristic CT features. Brain magnetic resonance imaging (MRI) revealed no abnormal lesions, whereas bone scintigraphy showed uptake in the right 10th rib (Figure 1C). Transbronchial lung cryo‐biopsy (TBLC) was performed, and CD1a and S100‐positive histiocytic cells were pathologically detected, indicating a diagnosis of PLCH (Figure 2).

Smoking cessation guidance and long‐term oxygen therapy were introduced. Although she quit smoking completely after guidance, she continued to be exposed to second‐hand smoke for a few months. Vital capacity (VC) and predicted diffuse capacity of the lung for carbon monoxide (%DLco) had declined to 3.09 L and 12.6% in January 2024. The 6 min walk distance (6MWD) decreased to 208 m, and transthoracic echocardiography revealed elevated right ventricular systolic pressure. In the subsequent right heart catheterisation (RHC), the mean pulmonary arterial pressure (mPAP) and pulmonary vascular resistance (PVR) were 41 mmHg and 8.8 Wood units, respectively, indicating severe pulmonary hypertension (PH). Sildenafil, a phosphodiesterase type 5 inhibitor, was initiated as treatment for PH.

**FIGURE 3:**
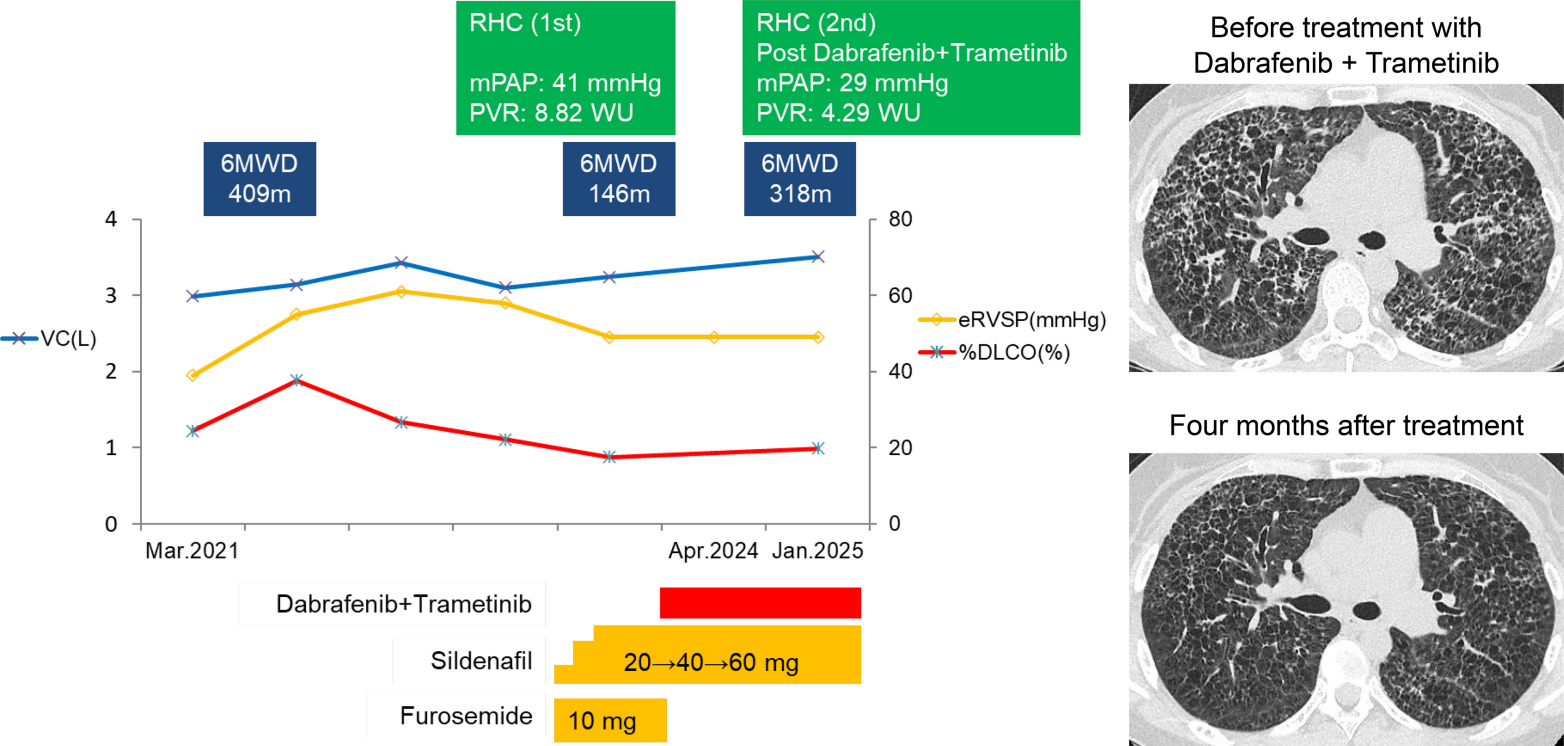
Clinical timeline illustrating the patient's response to treatment and major therapeutic interventions. RHC, right heart catheterisation; 6MWD, 6‐min walk test; mPAP, mean pulmonary arterial pressure; PVR, pulmonary vascular resistance; eRVSP, elevated right ventricular systolic pressure; %DLCO, diffuse capacity of the lung for carbon monoxide; VC, vital capacity.

Re‐analysis of lung biopsy specimens using PCR‐reverse sequence specific oligonucleotide method (MEBGEN BRAF kit) confirmed BRAF V600E mutation positivity, and combination therapy with dabrafenib and trametinib was initiated in April 2024. The patient developed grade 1 transient myalgia and fever; no other adverse events were observed. Chest CT three months after treatment showed improvement in the peri‐cystic opacity. Both radiological and symptomatic recovery was sustained for over 12 months. Pulmonary functional tests demonstrated improvement with VC to 3.51 L, %DLCO to 19.8%, and 6MWD extending to 312 m. RHC performed in January 2025 showed further haemodynamic improvement with mPAP (to 29 mmHg) and PVR (to 4.3 Wood units) (Figure 3). A follow‐up evaluation by bone scintigraphy is scheduled to be performed.

## 3 Discussion

In the pathogenesis of PLCH, smoking is thought to promote the recruitment of Langerhans cells to the lungs and to upregulate the production of inflammatory cytokines and chemokines, including tumour necrosis factor‐α (TNF‐α), transforming growth factor‐β, granulocyte‐macrophage colony‐stimulating factor, CCL20, and CCL7 [7]. Activating mutations in MAPK pathway genes, such as BRAF V600E and MAP2K1, promote the accumulation of Langerhans cells in the lung parenchyma [5]. In LCH mouse models overexpressing BRAF V600E, the production of CCL20 and CCL7 was elevated, and these chemokines may drive the migration of Langerhans and other inflammatory cells to the lungs. Inhibition of BRAF V600E signalling normalises CCL7 and CCL20 concentrations in bronchoalveolar lavage fluid [4], and there are many cases in which symptoms improve simply by quitting smoking. Corticosteroids suppress differentiation and proliferation of Langerhans cells by inhibiting the high expression of TNFα and Nuclear Factor kappa B pathway, thereby reducing T cell activation and the function of antigen presentation [8].

In a retrospective cohort study, 26 patients with BRAF V600–mutant LCH were treated with dabrafenib and/or trametinib, all of whom achieved sustained favourable responses [9]. Furthermore, in a phase I/II trial, with 12 patients with BRAF V600–mutant LCH receiving combination therapy with dabrafenib plus trametinib; the total response rate was 58.3% [10]. Although fever was the most frequent treatment‐related adverse event, it was tolerable in most cases. By contrast, in PLCH with BRAF mutations, only a single case demonstrating a favourable response to trametinib has been reported [11]. Most patients enrolled in clinical trials of BRAF/MEK inhibitors have mass‐forming lesions in the central nervous system, bone, or skin. Therefore, the therapeutic efficacy was assessed according to the Response Evaluation Criteria in Solid Tumours system, which is used for the therapeutic evaluation of solid tumours. However, there is no established method for evaluating therapeutic effects in patients with diffuse lesions, as in the present case. We assessed pulmonary function, exercise tolerance, and pulmonary hemodynamics before and after treatment and confirmed its efficacy. These assessments have not been previously reported, making this a valuable case.

PH is an independent marker of disease progression according to the Japanese severity classification system and a poor prognostic factor for PLCH [12]. PH often develops within 5 years of PLCH diagnosis [3]. Although a previous report showed that pulmonary vasodilators may prolong survival [13], their efficacy remains uncertain. BRAF/MEK signalling is known to promote the proliferation of vascular smooth muscle cells through activation of the MAPK pathway [14]. Therefore, the direct anti‐remodelling effects of BRAF/MEK inhibitors on pulmonary arteries may contribute to the improvement of PH. In the present case, BRAF/MEK inhibitors may be effective against both langerhans cells in the lung parenchyma and the abnormal proliferation of pulmonary arterial cells, ultimately leading to improved pulmonary function and haemodynamics.

TBLC is a widely used diagnostic technique that enables the collection of adequately sized lung specimens with minimal physical burden. Its diagnostic yield has been reported to be comparable to that of surgical lung biopsy in interstitial lung diseases, including PLCH. However, TBLC carries a risk of bleeding in patients with severe PH, and in such cases, surgical lung biopsy is recommended. In the present case, PH was suspected at the time of diagnosis; however, the estimated right ventricular systolic pressure by echocardiography was 40 mmHg, which indicated only mild elevation. Therefore, TBLC was judged safe to perform.

Clinical efficacy and safety of BRAF/MEK inhibitors for PLCH including the long‐term safety, duration of effect, tolerability of long‐term treatment, and risk of relapse remain unclear. Although further data are needed, dabrafenib plus trametinib appears to be an effective therapeutic option for PLCH with BRAF V600E‐mutant.

## Author Contributions

Akiko Sakurai, Chisato Tadokoro, Shunichi Kataoka, Taichi Miyawaki, and Motoyasu Kato were the attending physicians who treated the patient upon admission. Tetsutaro Nagaoka is an outpatient physician. Akiko Sakurai and Tetsutaro Nagaoka drafted the manuscript. Yuriko Terayama, Yuichi Nagata, NS, Takuo Hayashi, and Kazuhisa Takahashi assisted in writing the manuscript. Akiko Sakurai has submitted the final manuscript for publication. All authors have read and approved the final manuscript for submission.

## Consent

The authors declare that written informed consent was obtained for the publication of this manuscript and accompanying images and attest that the form used to obtain consent from the patient complies with the Journal requirements as outlined in the author guidelines.

## Conflicts of Interest

Kazuhisa Takahashi is an Editorial Board member of Respirology Case Reports and a co‐author of this article. He was excluded from all editorial decision‐making related to the acceptance of this article for publication. The other authors have no conflicts of interest to disclose.

## Data Availability

The data that support the findings of this study are available from the corresponding author upon reasonable request.
